# Cell Wall Composition Heterogeneity between Single Cells in Aspergillus fumigatus Leads to Heterogeneous Behavior during Antifungal Treatment and Phagocytosis

**DOI:** 10.1128/mBio.03015-19

**Published:** 2020-05-12

**Authors:** Robert-Jan Bleichrodt, Peter Foster, Gareth Howell, Jean-Paul Latgé, Nick D. Read

**Affiliations:** a Manchester Fungal Infection Group (MFIG), Infection, Immunity and Respiratory Medicine, The University of Manchester, Manchester, United Kingdom; b Probability and Statistics Group, School of Mathematics, The University of Manchester, Manchester, United Kingdom; c Manchester Collaborative Centre for Inflammation Research (MCCIR), Infection, Immunity, Inflammation and Repair, The University of Manchester, Manchester, United Kingdom; d Unité des Aspergillus, Département de Mycologie, Institut Pasteur, Paris, France; Universidade de Sao Paulo

**Keywords:** *Aspergillus fumigatus*, fungal diseases, single cell, cell heterogeneity, phenotypic heterogeneity, cell wall composition, antifungal tolerance, caspofungin, echinocandin, phagocytosis

## Abstract

Aspergillus fumigatus can cause a variety of lung diseases in immunocompromised patients, including life-threatening invasive aspergillosis. There are only three main classes of antifungal drugs currently used to treat aspergillosis, and antifungal resistance is increasing. Experimental results in fungal biology research are usually obtained as average measurements across whole populations while ignoring what is happening at the single cell level. In this study, we show that conidia with the same genetic background in the same cell population at a similar developmental stage show heterogeneity in their cell wall labeling at the single cell level. We present a rigorous statistical method, newly applied to quantify the level of cell heterogeneity, which allows for direct comparison of the heterogeneity observed between treatments. We show the extent of cell wall labeling heterogeneity in dormant conidia and how the level of heterogeneity changes during germination. The degree of heterogeneity is influenced by deletions of cell wall synthesizing genes and environmental conditions, including medium composition, method of inoculation, age of conidia, and the presence of antifungals. This heterogeneity results in subpopulations of germinating conidia with heterogeneous fitness to the antifungal caspofungin, which targets cell wall synthesis and heterogeneous sensitivity of dormant conidia to phagocytosis by macrophages.

## Abstract (precis)

The fungus Aspergillus fumigatus can cause invasive lung diseases in immunocompromised patients resulting in high mortality. Treatment using antifungal compounds is often unsuccessful. Average population measurements hide what is happening at the individual cell level. We set out to test what impact individual differences between the cell walls of fungal conidia have on their behavior. We show that a population of cells having the same genetic background gives rise to subpopulations of cells that exhibit distinct behavior (phenotypic heterogeneity). This cell heterogeneity is dependent on the strain type, gene deletions, cell age, and environmental conditions. By looking at the individual cell level, we discovered subpopulations of cells that show differential fitness during antifungal treatment and uptake by immune cells.

## INTRODUCTION

Heterogeneity within mold colonies originating from a conidium with a single nucleus can take on many forms from the heterogeneity in the morphology of different zones within the colony to the different hyphal compartments within single hyphae. Considerable heterogeneity has also been seen in the physiological activities in the different regions of a colony. For example, hyphae expressing and secreting high and low levels of glucoamylase (*glaA*) at the colony periphery of Aspergillus niger have been recognized (1). This hyphal physiological heterogeneity is promoted by septal plugging which prevents cytoplasmic mixing between adjacent hyphal compartments. Deletion of the gene encoding the septal pore plug protein HexA prevents plugging, and as a result, this mutant lacks cellular heterogeneity (2). This heterogeneity (wild type [WT]) or lack thereof (Δ*hexA*) was shown to impact heat resistance (3). Cell heterogeneity has also been linked to tolerance, heteroresistance, and persistence to antifungal drugs (4). Tolerance is defined as growth above the MIC (5), resulting from slow growing subpopulations of cells that show low levels of drug uptake (6), and is likely to be epigenetically modulated (7). Heteroresistance harbors cells that become resistant, e.g., by expressing proteins governing drug resistance, while others remain susceptible. Its mechanism has been reported to be of genetic, epigenetic, or nongenetic origins and as a result can be of a nonreversible or reversible nature (4, 8). Persistence is characterized by the emergence of a subpopulation of cells that become highly tolerant to the drug (9). Persistence can develop into resistance (nonreversible), when these cells acquire mutations in, e.g., drug target genes (10). However, the exact mechanisms linking single cell heterogeneity to this drug susceptibility status remain poorly understood, while they have an essential impact on the efficacy of drug treatment.

In this study, we investigated the relationships between conidial cell wall composition heterogeneity and susceptibility to the cell wall drug echinocandin caspofungin in the human opportunistic fungal pathogen Aspergillus fumigatus. We identified and analyzed subpopulations of conidia showing different patterns of heterogeneity at the cell wall level that manifest differential fitness to the antifungal caspofungin that targets cell wall synthesis and impact phagocytosis.

## RESULTS

### Cell wall composition is heterogeneous between single conidia, and this heterogeneity changes during germination.

**FIG 1:**
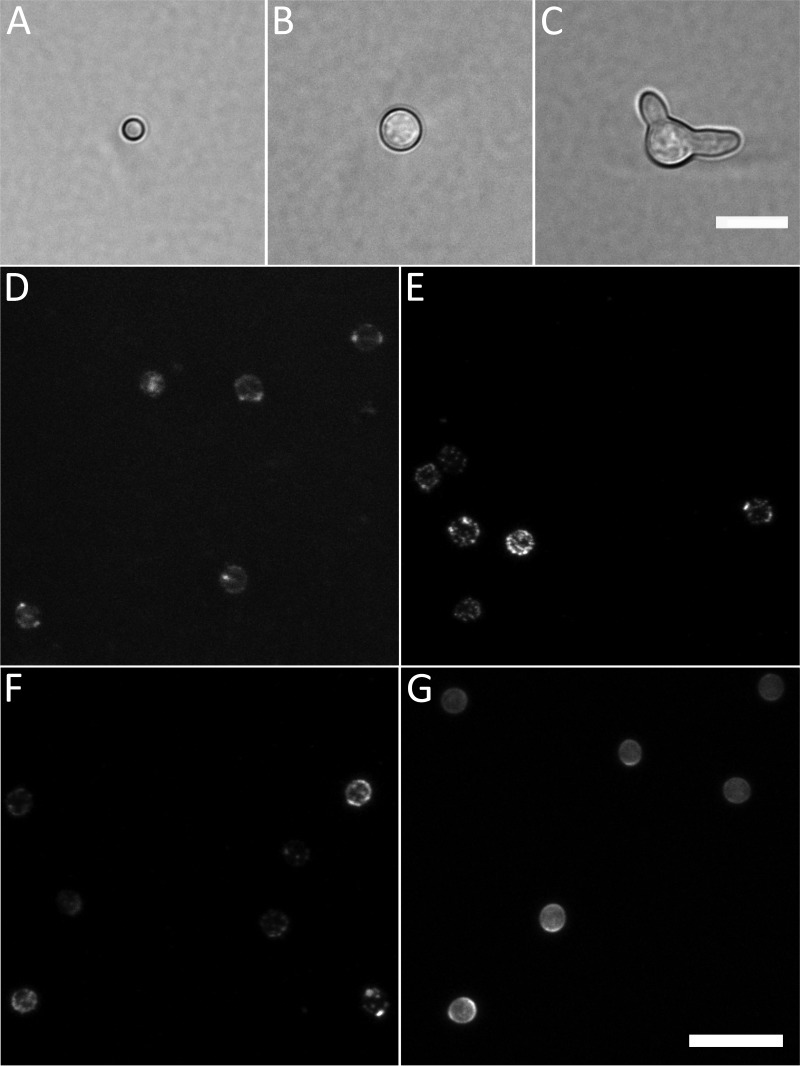
Cell morphotypes and labels used in this study. (A to C) The three developmental stages (morphotypes) analyzed in this study: dormant conidium (A), isotropically grown conidium (B), and germling (C) shown by bright-field microscopy. (D to G) Labeling of dormant conidia with fluorescently labeled lectins concanavalin A (ConA) (D), wheat germ agglutinin (WGA) (E), peanut agglutinin (PNA) (F), and Calcofluor White (CFW) (G). Summed intensity projections of Z-stacks are shown. Note the variation in fluorescent intensity between conidia stained with the same probe as well as the variability of the fluorescence intensity within single conidia. Bars, 10 μm (C and G).

In order to assess conidial heterogeneity, three cell types were analyzed: (i) 3-day-old dormant conidia (Fig. 1A), (ii) conidia that had undergone isotropic growth for 6 h at 37°C (swollen; Fig. 1B) and (iii) conidial germlings that had been incubated for 16 h at 30°C (germlings; Fig. 1C). Cell wall composition was assessed by confocal microscopy using fluorescently labeled lectins concanavalin A (ConA; binds mannose and glucose), wheat germ agglutinin (WGA; binds exposed chitin [GlcNAc]) and peanut agglutinin (PNA; binds galactose, and *N*-acetylgalactosamine) (11, 12) and the fluorescent brightener Calcofluor White (CFW; binds chitin and other insoluble polysaccharides [13]) (Fig. 1D to G, respectively). The lectins and CFW did stain the cell walls of all three conidial morphotypes of the wild-type (WT) strain CEA10.

**FIG 2:**
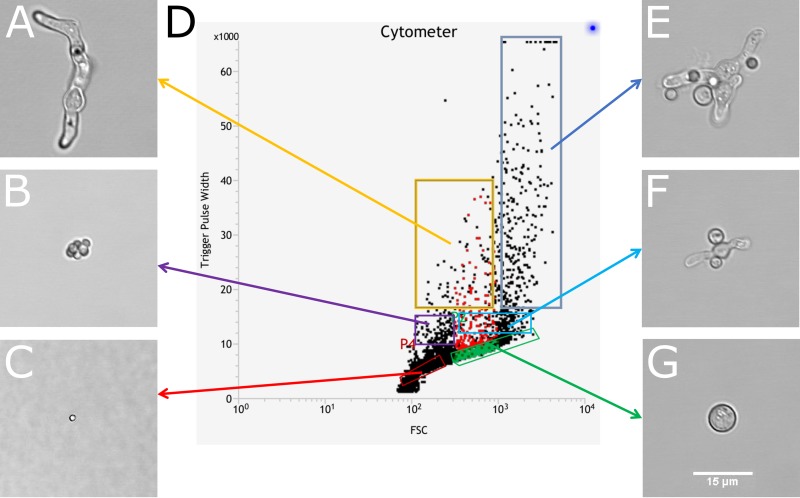
An overview of the trigger pulse width (TPW) against forward scatter (FSC) distributions of events found in a sample of cells incubated for 16 h at 30°C. The left set of images show a single germling (A), an aggregate of dormant conidia (B), and a single dormant conidium (C). The right set of images show a large aggregate of dormant and isotropically grown conidia mixed with germlings (E), a smaller aggregate of swollen conidia and germlings (F), and a single isotropically grown conidium (G). (D) Gates are indicated where these events are located in the flow cytometry plot. Bar = 15 μm (G).

**FIG 3:**
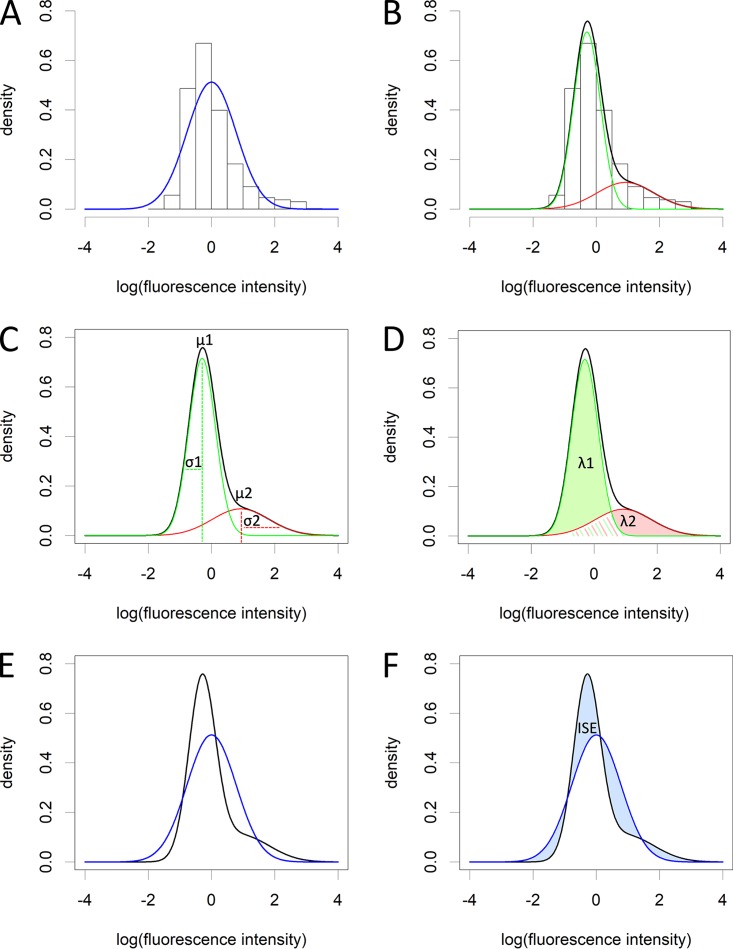
Level of heterogeneity explained. (A) First, the data are log transformed to reduce skewedness. Even after log transformation, the data (histogram) cannot be fitted with a single normal distribution (blue line). (B) Using the Bayesian information criterion (BIC) (see Materials and Methods) the number of subpopulations is determined. This calculation penalizes for adding additional subpopulations. In this study, all data could always be described by two subpopulations. The data are then fitted with a mixture of two normal distributions. Subpopulation 1 is indicated by a green line, subpopulation 2 by a red line, and the total fit, which is the sum of the two normal distributions is depicted with a black line. This fit can be described using three parameters: μ, σ (C), and λ (D) of each subpopulation, that represent the mean, standard deviation, and proportion of each subpopulation to the total population of cells, respectively. The area under each subpopulation fit is λ*_n_* (λ_1_ green and λ_2_ pink shaded areas under the curves in panel D). λ_1_ + λ_2_ = 1, since the sum represents the total population. (E) If we now plot the theoretical normal distribution from panel A (blue line) and the mixture normal distribution from panel B (black line), we can calculate the integrated squared error (ISE) (F) using μ, σ, and λ of each subpopulation (see Materials and Methods), which is basically the squared error of the surface area difference between the two lines (blue shaded areas between the curves) and indicates the level of heterogeneity. The data used to produce this figure are CFW-stained dormant conidia of strain CEA10 that had been grown for 3 days on minimal medium (MM) after spot inoculation to produce a radially extending colony.

**FIG 4:**
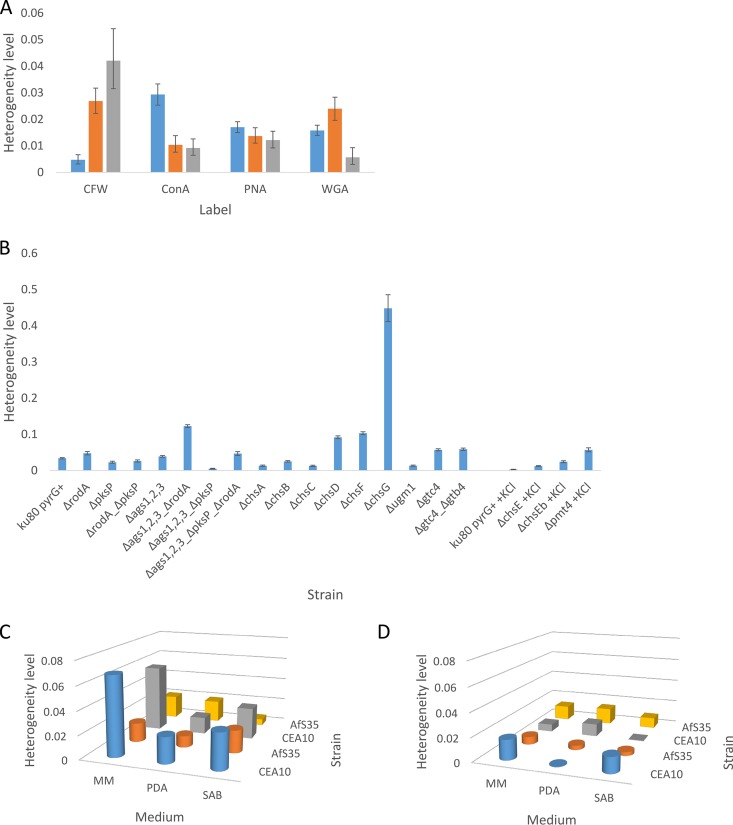
Heterogeneity of cell wall composition. (A) Heterogeneity of cell wall composition changes during germination. Dormant (blue bars), isotropically grown conidia (orange bars), and germlings (gray bars) were labeled with the fluorescent brightener Calcofluor White (CFW) or fluorescently labeled lectins concanavalin A (ConA), peanut agglutinin (PNA), or wheat germ agglutinin (WGA). For the number of cells observed, see Data Set S1 in the supplemental material (≥2 replicates). (B) Heterogeneity of CFW labeling in dormant conidia of cell wall synthesis gene mutants. The *ku80 pyrG*^+^ strain (with or without KCl) serves as a control strain. For the number of cells observed, see Data Set S1 (three replicates). Error bars represent 95% confidence intervals. (C and D) Heterogeneity of CFW labeling depending on strain, medium composition, and inoculation method after 3 days (C) and 7 days (D) of incubation at 37°C. Cylinders and boxes represent spot-inoculated and spread-inoculated conidia, respectively. See Data Set S1 for the number of cells observed and confidence intervals (three replicates).

All labels displayed heterogeneity between single cells that could each be characterized as two subpopulations of labeling patterns with regard to the total fluorescence per cell (see Fig. S1 in the supplemental material), as analyzed by flow cytometry (Fig. 2) and statistically determined by the Bayesian information criterion (BIC; Materials and Methods), which determines the best fit of the data by either a single normal distribution or a mixture of normal distributions. The level of heterogeneity was determined by calculating the integrated squared error (ISE) using mean, standard deviation, and lambda (proportion of mixing of the two normal distributions), of each of the subpopulations as parameters. This is explained step by step using an example in Fig. 3 (see also Data Set S1 in the supplemental material; Materials and Methods). An ISE value of 0 is computed when there is no heterogeneity at all in the cell population, since the single normal distribution fit (Fig. 3F, blue line) would then completely overlap with the fit of the mixture of normal distributions (Fig. 3F, black line). For example, a two times higher ISE corresponds to a twice higher level of cell heterogeneity, thus the ISE can be used to relatively compare heterogeneity levels of different cell populations. There was a large variance of heterogeneity observed depending on the label used and the developmental stage of the cells (Fig. 4A). CFW labeling heterogeneity was low in dormant conidia but gradually increased during germination. ConA labeling heterogeneity was first high in dormant conidia but then decreased during isotropic growth and was kept constant during germ tube formation. PNA labeling heterogeneity was similar across all developmental stages. WGA labeling heterogeneity first increased during swelling of conidia but then greatly decreased after germ tube formation. Among dormant conidia, the highest heterogeneity levels were observed for ConA; for swollen conidia, CFW and WGA; and for germlings with CFW labeling. Our data indicate that the fungal cell wall is a highly heterogeneous and dynamic structure with its level of heterogeneity being specific to its stage of cell development.

10.1128/mBio.03015-19.1 FIG S1 Fits of a mixture of two normal distributions of log-transformed fluorescence intensities of fluorescently labeled fungal cells of the combined biological replicates. These graphs correspond to the heterogeneity data presented in Fig. 4. Log-transformed data are represented by the histogram. The distributions of the subpopulations are shown by the red and green lines. The fit of the mixture of distributions is indicated by a black line, while the theoretical fit of one normal distribution is indicated by a blue line. The deviation of the black and blue lines is indicative for the heterogeneity level (ISE). The parameters of the fits can be found in Data Set S1. Download FIG S1, TIF file, 2.8 MB. Copyright © 2020 Bleichrodt et al. 2020 Bleichrodt et al. This content is distributed under the terms of the Creative Commons Attribution 4.0 International license.

10.1128/mBio.03015-19.10 DATA SET S1 Parameters and their confidence intervals resulting from the statistical analysis of heterogeneity. Download Data Set S1, XLSX file, 0.04 MB. Copyright © 2020 Bleichrodt et al. 2020 Bleichrodt et al. This content is distributed under the terms of the Creative Commons Attribution 4.0 International license.

### Mutations in cell wall synthesizing genes affect cell wall labeling heterogeneity.

Dormant conidia of cell wall mutants, defective in one or more cell wall synthesizing genes, were labeled with CFW and subjected to flow cytometry. Conidia of Δ*pksP*, *ΔrodA_ΔpksP*, Δ*ags123_*Δ*pksP*, Δ*chsA-C*, and *Δugm1* mutant strains (Table 1) showed lower CFW labeling heterogeneity levels than the control (*ku80 pyrG*^+^), whereas Δ*rodA*, Δ*ags1*,*2*,*3*_Δ*rodA*, Δ*ags1*,*2*,*3*_Δ*pksP*_Δ*rodA*, Δ*chsD-G*, Δ*gtc4*, Δ*gtc4*_Δ*gtb4*, and *Δpmt4* mutants showed higher heterogeneity levels than their respective controls (*ku80 pyrG*^+^ and *ku80 pyrG*^+^ plus KCl) (Fig. 4B; Fig. S1; Data Set S1). The Δ*ags123* mutant showed levels similar to those of the control. Among all mutants tested, Δ*chsE*, Δ*chsEb*, Δ*chsG*, and Δ*pmt4* mutants showed >4 times higher heterogeneity levels than the control strain, while most other mutants never reached higher than twice the heterogeneity level than that of the control. All mutants showed two partly overlapping subpopulations, except the Δ*ags1,2,3*_Δ*rodA* mutant which showed distinct mostly nonoverlapping subpopulations (Fig. S1).

**TABLE 1:** Strains used in this study

Strain	Relevant genotype	Function(s)^*a*^	Reference strain or reference
CEA10	WT		FGCS A1163
CEA17	*pyrG* mutant	Pyrimidine biosynthesis	FGSC 1152
AfS35	Δ*akuA*::*loxP*	Nonhomologous end joining	FGCS A1159
*ku80 pyrG*^+^ (A1160 *pyrG*+)	Δ*ku80 pyrG*^+^	Nonhomologous end joining	39
Δ*rodA*	Δ*ku80* Δ*rodA*	Rodlet formation	40
Δ*pksP*	Δ*ku80* Δ*pksP*	Melanin production	41
Δ*rodA-pksP*	Δ*ku80* Δ*rodA* Δ*pksP*	Rodlet formation, melanin production	42
Δ*ags123*	Δ*ku80* Δ*ags1* Δ*ags2* Δ*ags3*	α-Glucan synthesis	43
Δ*ags123-rodA*	Δ*ku80* Δ*ags1* Δ*ags2* Δ*ags3* Δ*rodA*	α-Glucan synthesis, rodlet formation	41
Δ*ags123-pksP*	Δ*ku80* Δ*ags1* Δ*ags2* Δ*ags3* Δ*pksP*	α-Glucan synthesis, melanin production	41
Δ*ags123-rodA-pksP*	Δ*ku80* Δ*ags1* Δ*ags2* Δ*ags3* Δ*rodA* Δ*pksP*	α-Glucan synthesis, rodlet formation, melanin production	41
Δ*GTC4*	Δ*ku80* Δ*GTC4*	GAG production	L. Muszkieta, T. Fontaine, J. P. Latgé, unpublished
Δ*GTC4-GTB4*	Δ*ku80* Δ*GTC4* Δ*GTB4*	GAG production	L. Muszkieta, T. Fontaine, J. P. Latgé, unpublished
Δ*pmt4*	Δ*ku80* Δ*pmt4*	Mannosyltransferase	44
Δ*ugm1*	Δ*ku80* Δ*ugm1*	UDP-galactopyranose mutase	45
Δ*chsA*	Δ*ku80* Δ*chsA*	Chitin synthase	46
Δ*chsB*	Δ*ku80* Δ*chsB*	Chitin synthase	46
Δ*chsC*	Δ*ku80* Δ*chsC*	Chitin synthase	46
Δ*chsD*	Δ*ku80* Δ*chsD*	Chitin synthase	46
Δ*chsE* (*csmA*)	Δ*ku80* Δ*chsE*	Chitin synthase	47
Δ*chsEb* (*csmB*)	Δ*ku80* Δ*chsEb*	Chitin synthase	47
Δ*chsF*	Δ*ku80* Δ*chsF*	Chitin synthase	46
Δ*chsG*	Δ*ku80* Δ*chsG*	Chitin synthase	46
MFIGRag29	P*gpdA*::*katushka*::T*trpC pyrG*+	Red fluorescent protein	48

a GAG, galactosaminogalactan.

### Environmental conditions affect cell heterogeneity within the same genetic background and between strains.

We tested whether inoculation method, strain, medium, and culture age had an effect on the heterogeneity level of populations of dormant conidia that were stained with CFW. Conidia of strain CEA10 or AfS35 were inoculated by evenly spreading them (spread) or applying a 2-μl spot in the center of the culture (spot) on minimal medium (MM), potato dextrose agar (PDA), or Sabouraud (SAB) medium. Cultures were incubated for 3 or 7 days. After 3 days of growth, strain CEA10 grown on MM showed highest heterogeneity levels, followed by SAB and PDA for both spread and spot inoculation methods (Fig. 4C; Fig. S1; Data Set S1). Spot inoculation gave higher heterogeneity levels than spread inoculation for strain CEA10 on all media tested, except on SAB. The reverse was true for AfS35 which is lacking the *ku80* gene, except on SAB. For strain AfS35, growth on SAB generated the highest heterogeneity during spot inoculation followed by MM and PDA, whereas for spread inoculation, both MM and PDA showed higher levels than SAB.

When cultures were grown for 7 days, they generally showed lower heterogeneity levels than 3-day-old cultures on the same medium (Fig. 4D). Differences in heterogeneity levels between treatments were also less pronounced compared to 3-day-old cultures. For CEA10 spot inoculation, MM and SAB still exhibited some heterogeneity; however, on PDA, almost none was observed. For AfS35 spot inoculation, PDA and SAB showed similar low heterogeneity, while that on MM was higher than those. CEA10 spread inoculation showed again lower heterogeneity levels than spot inoculation on MM and SAB, except for PDA, for which the reverse was true. For CEA10 spread inoculation on PDA, MM and SAB showed descending heterogeneity levels. For strain AfS35, all media showed higher heterogeneity levels after spread inoculation compared to spot inoculation. For AfS35 spot inoculation, PDA and SAB showed similar but lower levels than MM. For AfS35 spread inoculation, MM and PDA showed similar levels but higher than the level on SAB. When conidia were allowed to develop on MM plus caspofungin for 3 days from spread inoculation, we observed that heterogeneity levels decreased compared to growth on MM. Strains CEA10 and AfS35 showed ISEs of 0.057 (bootstrap 95% confidence interval [CI], 0.053 to 0.061) and 0.020 (CI, 0.018 to 0.022) on MM, while on MM plus caspofungin, ISEs of 0.033 (CI, 0.030 to 0.036) and 0.015 (CI, 0.014 to 0.017) were observed, respectively (Data Set S1 and Fig. S1).

### The observed cell heterogeneity is not caused by heterogeneity within the inoculum.

To test whether the observed cell heterogeneity relates to heterogeneity within the multiconidia inoculum, we isolated a single conidium on minimal agar medium by fluorescence-assisted cell sorting (FACS) in duplicate. We allowed these cells to develop sporulating colonies and compared them to a colony that was derived from spot inoculation with multiple isogenic conidia. Conidia were harvested and stained with CFW, and their fluorescence intensities were measured using flow cytometry. Although these newly developed conidia had originally been derived from a single conidium inoculum, we still observed two subpopulations and heterogeneity levels of CFW labeling (ISE = 0.0021 and CI of 0.0009 to 0.0041; ISE = 0.0024 and CI of 0.0011 to 0.0045) similar those of conidia derived from a colony that had been inoculated with multiple conidia (ISE = 0.0026 and CI of 0.0014 to 0.0041). This indicates that the observed conidial heterogeneity is driven by intrinsic factors and not by phenotypically inherited differences in the pattern of CFW staining of chitin in the cell wall.

### Cell wall composition heterogeneity of conidia confers fitness during caspofungin treatment.

**FIG 5:**
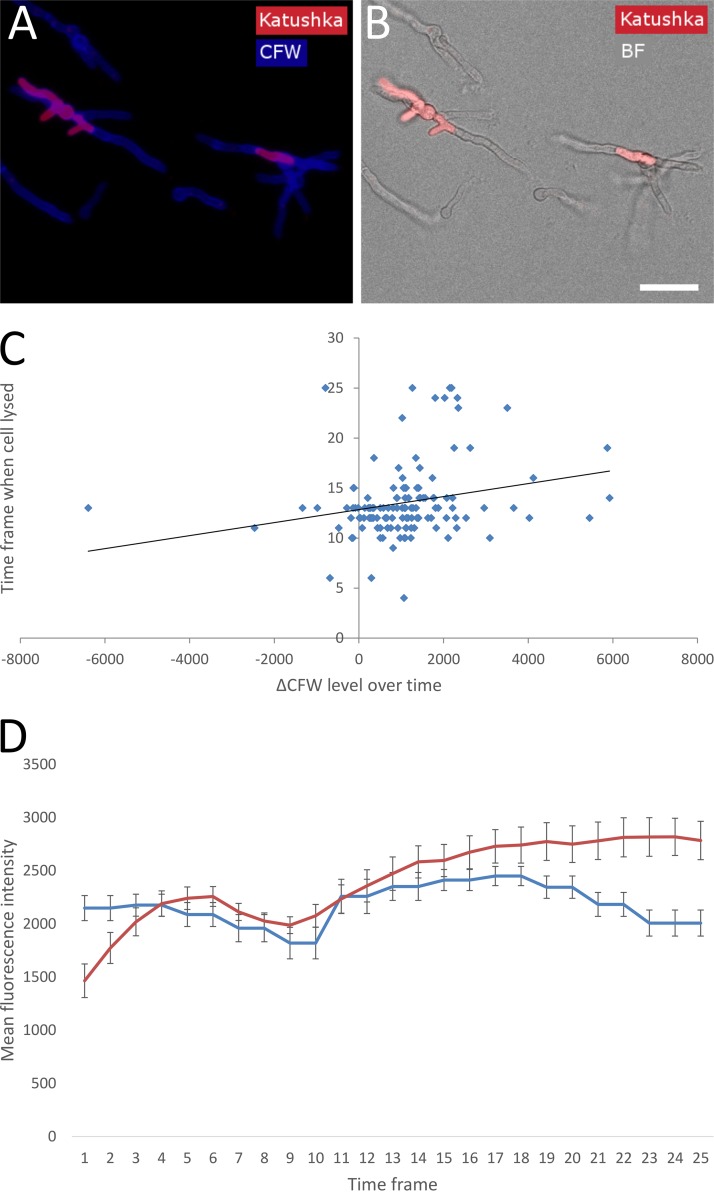
Relation of CFW labeling and survival of germinating conidia on caspofungin. (A and B) Conidia expressing cytosolic Katushka (strain MFIGRag29) were germinated in the presence of CFW (25 nM) and caspofungin (0.5 μg/ml). Snapshots are shown after 15 h of incubation at 37°C. Bar = 20 μm (B). Entire cells can be seen by CFW labeling (A) or bright-field microscopy (B). Living compartments are red, and lysed compartments have lost red fluorescence. (C) Correlation between CFW labeling increase (ΔCFW level = ending CFW level − beginning CFW level) and time frame when a cell had lysed. Correlation = 0.24, *n* = 128, power = 0.8, *P* = 0.05. A trendline is drawn to show the positive correlation. (D) Average fluorescence intensity of the total population of cells during germination on MM (control; blue line) and MM plus caspofungin (red line) (*n* = 115 and 136, respectively). Error bars indicate 95% confidence intervals. Time frame intervals are ∼30 min.

Cell wall heterogeneity could have a fitness benefit when environmental conditions change. In this case, cells that exhibit a higher level of a beneficial trait or a lower level of an undesired trait may have a higher fitness than others. We studied the behavior of single cells when grown in the presence of caspofungin using time-lapse confocal laser scanning microscopy (CLSM). To this end, conidia expressing the cytosolic red fluorescent protein Katushka (strain MFIGRag29; Table 1) were allowed to germinate in liquid MM with or without the presence of 0.5 μg/ml caspofungin. CFW was added at *t* = 0, at very low concentrations (25 nM), to monitor the chitin level of the cell walls of individual cells over time. Z-stacks of images were captured at ∼30-min intervals after immersion in the appropriate medium for 1 h (this was the time required to set up the microscope for automated imaging). Cytosolic Katushka was monitored to visualize and quantify cell lysis (the cell compartments lose their red fluorescence upon lysing) (Fig. 5A and B; see also Movie S1 in the supplemental material). Bespoke scripts in ImageJ were produced to determine the summed CFW fluorescence intensity in Z of individual cells over time. In the absence of caspofungin (control condition), it took ∼8 h to develop germlings, whereas for the caspofungin-treated condition, it took ∼15 h to reach the same developmental state. Out of the 664 cells measured, 13.6% (±3.8%; 95% confidence interval) of the cells survived caspofungin treatment. Five different types of behavior were exhibited by different cell subpopulations within the cell population as a whole. (i) Cells underwent lysis during swelling (1.5% ± 0.23%) or (ii) cells lysed after germ tube formation (81.2% ± 3.0%; Movie S1) starting from ∼7 h and died as a result, indicated by loss of Katushka fluorescence and no observed regrowth. (iii) Cells lysed partly, but at least one hyphal compartment survived, then continued to grow, but finally died through subsequent lysis (3.7% ± 0.80%; Movie S2). (iv) Cells lysed one or more times but always recovered by regrowing (12.5% ± 1.9%; Movie S3). (v) Cells germinated at later time points and never lysed during the time window imaged (1.1% ± 2.1%). In the control condition, no cell lysis was observed (Movie S4).

10.1128/mBio.03015-19.5 MOVIE S1 Two cells cytosolically expressing Katushka lyse after germ tubes have been formed during caspofungin treatment. Download Movie S1, AVI file, 0.03 MB. Copyright © 2020 Bleichrodt et al. 2020 Bleichrodt et al. This content is distributed under the terms of the Creative Commons Attribution 4.0 International license.

10.1128/mBio.03015-19.6 MOVIE S2 A germling partly lyses two times before finally lysing to death during caspofungin treatment. Download Movie S2, AVI file, 0.03 MB. Copyright © 2020 Bleichrodt et al. 2020 Bleichrodt et al. This content is distributed under the terms of the Creative Commons Attribution 4.0 International license.

10.1128/mBio.03015-19.7 MOVIE S3 Germling lysing one or more times, but it recovers by regrowing during caspofungin treatment. Download Movie S3, AVI file, 0.04 MB. Copyright © 2020 Bleichrodt et al. 2020 Bleichrodt et al. This content is distributed under the terms of the Creative Commons Attribution 4.0 International license.

10.1128/mBio.03015-19.8 MOVIE S4 Cells germinating during control treatment do not show lysis. Download Movie S4, AVI file, 0.02 MB. Copyright © 2020 Bleichrodt et al. 2020 Bleichrodt et al. This content is distributed under the terms of the Creative Commons Attribution 4.0 International license.

We set out to investigate why some cells lysed and died, and other cells survived during caspofungin treatment. Strain CEA17 was germinated in the presence of MM plus uridine to complement its *pyrG* deficiency for 16 h at 30°C. Then medium was changed for MM without uridine to inhibit growth. Killing of cells by caspofungin was investigated with propidium iodide labeling, and time-lapse imaging was performed for 16 h. These isotropically swollen cells and germlings never did lyse (data not shown). This shows that normally growing cells are required for caspofungin to cause cell lysis.

We also looked into the relationship between CFW staining levels and caspofungin tolerance at the single cell level. There was a positive correlation (0.24; *P* = 0.05) with the CFW labeling increase over time and survival time of individual cells (Fig. 5C). Moreover, the mean fluorescence intensity of the total population during caspofungin treatment increased over time and was higher than in the control condition later on (Fig. 5D), indicating a coping strategy to accommodate the loss of β-glucan in the cell wall by producing more chitin. In contrast, the overall fluorescence intensity was relatively stable over time in the control situation (Fig. 5D). Overall, these data indicate that a majority of the cells are sensitive to caspofungin treatment, but during germination, a robust subpopulation emerges that develops tolerance to caspofungin, correlating with increased chitin levels (Fig. 5C), as visualized by CFW labeling.

To test whether this behavior is a case of tolerance, heteroresistance, or resistance, conidia were pregrown on MM with caspofungin, and were subsequently grown on caspofungin again to count colony-forming units (CFU) and on MM as a control. The same was done for conidia pregrown on MM. We found that conidia that were pregrown on caspofungin did not show a higher CFU ratio (CFU on caspofungin/CFU on MM) than conidia pregrown on MM. This shows that the heterogeneous behavior observed above is not due to selection of a (hetero)resistant subpopulation on caspofungin but can be classified as tolerance.

### Dormant conidia exhibit heterogeneity in their susceptibility to phagocytosis by macrophages.

**FIG 6:**
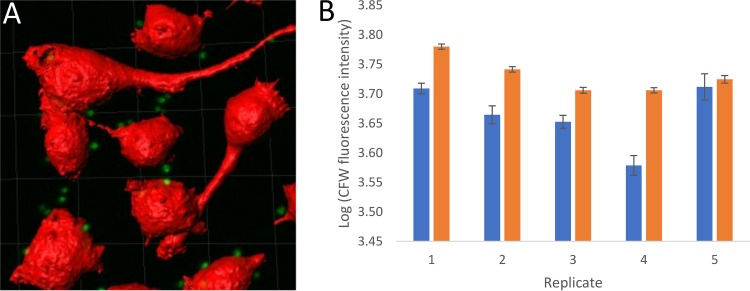
Heterogeneous phagocytosis of CFW-labeled conidia. (A) Imaris image analysis. Macrophages segmented as surfaces (shown in red; CellMask Deep Red) and conidia segmented as spots (shown in green; cytosolic Katushka). The conidia within each macrophage were counted by using the Spots Split Into Surface Objects XT function on the segmented spots. (B) CFW level (log intensity summed) of internalized (orange bars) versus noninternalized conidia (blue bars). Replicates 1 to 4 show a significant difference, but replicate 5 does not. Error bars indicate 95% confidence intervals.

To test whether phagocytosis is impacted by conidial heterogeneity, RAW264.7 macrophages were challenged with dormant conidia of strain MFIGRag29 expressing cytosolic Katushka (Table 1), either prelabeled with CFW or not, and fixed after 1 h. Macrophage cells were stained with CellMask Deep Red to visualize their plasma membranes, and Z-stacks were produced with CSLM. Using Imaris image analysis software, macrophages were segmented as surfaces (red), and conidia (green) were segmented as spots in three dimensions (3D) (Fig. 6A). Using the “Spots Split Into Surface Objects” function, conidia that were internalized were discriminated from the noninternalized ones (see Materials and Methods). We observed that the subpopulation of internalized conidia had a significantly higher CFW labeling intensity than the subpopulation of noninternalized conidia for 4/5 replicates tested (Fig. 6B). Similar phagocytosis rates (*P* = 0.78) of 83.9% ± 1.4% and 84.6% ± 4.3% were observed for unlabeled and CFW-labeled conidia (MFIGRag29), respectively, indicating that CFW labeling does not affect the phagocytosis rate of the total population of conidia.

## DISCUSSION

### Cell heterogeneity.

Experimental results in research are commonly represented as average measurements across whole populations of cells while ignoring what is happening at the single cell level within these populations. For example, it may be that two populations have the same average, but that one is more heterogeneous than the other and therefore exhibits a higher survival rate during adverse environmental conditions (see also reference 14).

In the present study, we set out to analyze the heterogeneity of characteristics of individual cells and observed how this resulted in unique patterns of cell behavior. We found that the conidial cell wall is heterogeneous in composition and that this heterogeneity changes during germination. For each cell wall label, we observed two subpopulations, i.e., one with a low and one with a high fluorescence intensity phenotype (Fig. 3; see also Fig. S1 in the supplemental material). We could not attribute a single cell to a specific subpopulation with 100% certainty, since subpopulations were partly overlapping. Therefore, each cell has a probability that it either belongs to the first or second subpopulation. It is thus impossible to say that when, e.g., a dormant conidium belongs to the high CFW subpopulation, that it also belongs to the high ConA, PNA, and WGA subpopulations in a population of conidia that would have been simultaneously stained with all four labels. However, if we look at the proportions (lambda) of the subpopulations of each cell wall staining, we can see that they are not the same: CFW lambda1 = 0.165, ConA lambda1 = 0.955, PNA lambda1 = 0.942, and WGA lambda1 = 0.934 (see Data Set S1 in the supplemental material). This indicates that most conidia belong to the subpopulation having the low fluorescent ConA, PNA, and WGA phenotype. In contrast, most cells (lambda2 = 1 − lambda1 = 1 − 0.165 = 0.835) belong to the highly fluorescent CFW phenotype, but this lambda is not equal to the low fluorescent phenotype of the other labels. This suggests that a small proportion (0.165 × 0.955 × 0.942 × 0.934 = 0.139) of the conidia exhibit the low fluorescent phenotype of all labels. However, there are still minor subpopulations of cells having all other of the remaining 2^4^ = 16 possible combinations (for two phenotypes [low or high fluorescence intensity] and four labels).

Heterogeneity can be a result of the cell cycle, cell ageing, epigenetic regulation (reviewed in reference 15), cellular metabolism (16), and gene expression stochasticity (17). This stochasticity itself can also be regulated (18). The fact that CFW labeling levels of individual conidia were not inherited in their offspring is in line with this. We observed that both genes that are directly involved in chitin synthesis, as well as genes that are involved in the production of other cell wall polymers, affected the level of chitin heterogeneity as visualized by CFW staining (Fig. 4B). Moreover, medium composition, culture age, inoculation method (Fig. 4C and D), and caspofungin all affected cell wall CFW labeling heterogeneity. When colonies were incubated for 3 or 7 days at 37°C, overall, we observed markedly lower heterogeneity levels for the latter condition. We hypothesize that this is due to conidial maturation. After 3 days, strain CEA10 showed generally higher heterogeneity levels than strain AfS35 (Fig. 4C). Possibly, AfS35 has faster maturation of conidia. This is indicated by the observation that AfS35 generally showed less change in heterogeneity between 3 and 7 days incubation compared to CEA10 (Fig. 4C and D). We observed lower heterogeneity levels for conidia produced in the presence of caspofungin compared to the control. Recently, it was shown that the cell wall integrity pathway (CWIP) that is the main signaling route that controls new formation of cell wall is involved in conidiogenesis (19). Moreover, CWIP is involved in caspofungin tolerance (20, 21). It may well be that the observed heterogeneity changes for conidia formed in the presence of caspofungin relate to the CWIP. Heterogeneity in conidial survival has also been observed during heat treatment (22, 23), oxidative stress, and UV radiation (23). Temperature fluctuation (23), water activity and pH during conidiation (24), and conidia maturation (25) are factors that could give rise to this phenotypic heterogeneity. This is in line with our observations that environmental factors such as inoculum method, medium composition, and caspofungin treatment all resulted in differential heterogeneity levels.

Together, these results provide insights into the causal complexity in cell wall synthesis that generates the different patterns of heterogeneity in cell wall composition. The observed heterogeneity of cell wall composition may have an effect on single cell behavior on all processes where the cell wall is involved. This can be, for example, the secretion of carbohydrate degrading enzymes, export of extracellular vesicles, virulence, and/or tolerating adverse environmental conditions. We have shown, that single cells of A. fumigatus exhibit heterogeneity in antifungal tolerance and susceptibility to phagocytosis (see below).

### Heterogeneity and antifungal tolerance.

It has previously been reported that caspofungin treatment increases cell wall chitin content on a population level (26, 27). We observed heterogeneity of CFW labeling and therefore set out to investigate single cell caspofungin tolerance in relation to CFW labeling intensity. We could not find a correlation between initial CFW fluorescence intensity in dormant conidia and CFW levels during germination (data not shown). However, we observed a positive correlation for the CFW level increase over time with survival time at the single cell level using time-lapse microscopy. Cells that were delayed in germination were tolerant to caspofungin. Moreover, the growth rate of cells just before cell death occurred was negatively correlated with the change in CFW level over time (data not shown). Together this indicates that slow growing cells are better in tolerating caspofungin treatment, possibly due to having a better ability of increasing their cell wall chitin content than fast growing cells. This is consistent with nongrowing or slow growing bacteria that tolerate both complement and antibiotics (28–30) and that slow growing A. fumigatus strains are killed less efficiently *in vivo* than faster growing strains (31). Germinating cells that were inhibited in growth did not lyse in the presence of caspofungin (CEA17 without uridine), indicating that only normally growing cells are susceptible to caspofungin. When we looked at the individual cell level (MFIGRag29), most cells (82.7%) lysed during caspofungin treatment, whereas small subpopulations initially survived and then lysed (3.7%) or survived (13.6%).

In microcolonies growing in the presence of caspofungin, hyphal lysis was observed only in apical compartments that then continued growth by intrahyphal extension of a new hyphal tip (32). We found that most conidia (86.4%) that were germinating in the presence of caspofungin died before able to form such an adapted continuously regenerating microcolony. Indeed, significantly more germlings survived after they had formed at least one septum than died after this time point. The importance of septa is exemplified by the fact that the *rho4* and *hexA* mutants that do not produce septa and cannot close septa, respectively, showed more extensive hyphal lysis during caspofungin treatment than the wild type (33).

Since invasive fungal infections of the lungs are always discovered after inhaled conidia have already produced established fungal colonies in the host, caspofungin treatment can only delay further fungal colonization at this stage. However, patients that are expected to develop invasive fungal infections, due to, e.g., bone marrow transplantation, might possibly greatly benefit from prophylaxis with caspofungin, which is able to kill most of the inhaled conidia when germinating. Indeed, caspofungin prophylaxis has been shown to display effectiveness in preventing invasive fungal aspergillosis after stem cell transplantation (34). Still, in our study, 13.6% of cells survived caspofungin treatment. Cell heterogeneity may therefore be one of the key factors explaining why antifungal treatment often is unsuccessful. Simultaneous treatment with multiple antifungals might provide a way to attack the heterogeneous population of cells. Alternatively, novel drugs could be screened for that diminish or lower the level of heterogeneity in the fungal cell population and could be used in combination with caspofungin treatment to effectively kill all fungal cells. In the inhibition zone of fluconazole disc diffusion tests, growing C. albicans colonies were observed. These colonies were diminished when fluconazole was used in combination with a range of adjuvants (6). This strengthens our hypothesis that combination therapy could be more effective than single antifungal treatment alone.

### Conidial heterogeneity and phagocytosis.

We observed that the subpopulation of internalized conidia had a significantly higher CFW labeling level than the subpopulation of noninternalized conidia for 4 out of 5 replicates tested. Both unlabeled and CFW-labeled conidia showed similar levels of internalization. Therefore, the observed difference in phagocytosis rate between the low- and high-CFW-stained conidial subpopulations could not be due to blocking of antigens by CFW labeling or CFW being an antigen itself. Chitin is an immune-stimulating agent (35) and can be labeled by CFW. It may be that the subpopulation of conidia with high CFW levels was therefore more readily detected by the macrophages than the subpopulation having low CFW levels. Once conidia have been phagocytosed, they are protected from killing by neutrophils, by inhibiting germination, and thereby promoting persistence (31). Therefore, future research should focus on whether the heterogeneity within conidial populations we have observed results in the persistence of specific conidial subpopulations *in vivo*.

## MATERIALS AND METHODS

### Strains.

See Table 1 for all strains used in this study.

### Growth conditions.

To obtain fresh conidia, Aspergillus fumigatus was routinely grown in 25-cm^2^ culture flasks containing 10 ml of 4% potato dextrose agar (PDA) and grown at 37°C for 3 days, unless stated otherwise. Conidia were either inoculated by spreading 20 μl conidial suspension evenly on the medium with a loop or by inoculating with a 2-μl drop of conidial suspension in the middle of the medium. Conidia were harvested by adding 10 ml saline Tween (ST) (0.9% NaCl plus 0.005% Tween 80) to culture flasks that were shaken vigorously. In the case of problems encountered suspending conidia (e.g., with melanin mutants), conidia were collected in ST by scraping with a loop. The Δ*chsE*, Δ*chsEb*, and Δ*pmt4* mutants needed to be grown on PDA supplemented with 6% KCl, since they showed no or little sporulation on PDA. Therefore, the corresponding control *ku80 pyrG*^+^ strain was also grown on PDA plus 6% KCl as a control for these strains. Conidial suspensions were filtered through 40- to 100-μm filters (EASYstrainer; Greiner Bio-One).

To obtain isotropically grown conidia or germlings, 10^8^ conidia were inoculated in 25-cm^2^ culture flasks containing 20 ml liquid minimal medium (MM) plus 1% glucose and incubated for 6 h at 37°C or 16 h at 30°C, respectively, as standing cultures. Cells were harvested by shaking and vortexing, and cultures were filtered through 100-μm filters (EASYstrainer; Greiner Bio-One).

### Fluorescent labeling of conidia and monitoring CFW and Katushka fluorescence during caspofungin treatment.

Fluorescent labeling of conidia and monitoring CFW and Katushka fluorescence during caspofungin treatment are described in Text S1 in the supplemental material.

10.1128/mBio.03015-19.9 TEXT S1 Supplemental Materials and Methods. Download Text S1, DOCX file, 0.02 MB. Copyright © 2020 Bleichrodt et al. 2020 Bleichrodt et al. This content is distributed under the terms of the Creative Commons Attribution 4.0 International license.

### Flow cytometry. (i) Gating strategy: obtaining single cells of dormant conidia, isotropically grown conidia, and germlings.

Flow cytometry and fluorescence-assisted cell sorting (FACS) were set up to measure and isolate single dormant or isotropically grown conidia and germlings. The A. fumigatus wild-type strain CEA10 was grown on potato dextrose agar (PDA) medium for 3 days at 37°C, and dormant conidia were harvested in ST. Conidial suspensions were either filtered through a 5 μm filter (Minisart) or not filtered and passed through the flow cytometer. First, the side scatter (SSC) and forward scatter (FSC) of cells were measured. SSC measures cell complexity, and FSC measures cell size (36). Most events were observed in an oval-shaped region indicated by the black gate (see Fig. S2A in the supplemental material). Cells within and outside this gate were sorted on slides and checked by bright-field microscopy. All cells outside the gate were aggregates of two or more conidia, whereas most events within the gate were single conidia but still contained some doublets. To obtain single cells, we passed the conidia through a 5-μm filter (Fig. S2B). This step removed most of the cell aggregates and was used to draw the single cell gate with more precision (Fig. S2A and B). Since untreated cells still contained some doublets in the single cell gate, we also included trigger pulse width (TPW) as a selection criterion, which measures the signal width of the light pulse above the threshold. Usually, the more elongated a cell is, the higher is its TPW. All cells of the filtered conidia were contained in a rectangular gate (Fig. S2D); some of the untreated cells, however, fell outside this gate (Fig. S2C). We confirmed by microscopy that those events were doublets of cells and that the single cell gate (Fig. S2C and D) almost exclusively contained single dormant conidia. In total, ∼75% of the untreated sample contained single dormant conidia.

10.1128/mBio.03015-19.2 FIG S2 Flow cytometry of conidia directly harvested from the colony (A and C) and conidia passed through a 5-μm filter to remove aggregates (B and D). Side scatter (SSC) and forward scatter (FSC) (A and B) and trigger pulse width (TPW) and FSC (C and D) were measured to analyze the cells. Rainbow colors show frequency of events with red showing the most events and blue showing the least events. Panels C and D show daughter gates of panels A and B, respectively. Any doublets or aggregates of conidia show up above the gate drawn in panel C. Filtering of conidia removes these cells from the population (D). Percentage of cells in each gate expressed to the total population (A and B) or mother gate (C and D) are indicated. The sorting efficiency of single cells as verified by microscopy was 98%. Download FIG S2, TIF file, 1.1 MB. Copyright © 2020 Bleichrodt et al. 2020 Bleichrodt et al. This content is distributed under the terms of the Creative Commons Attribution 4.0 International license.

We next set out to find where isotropically grown conidia could be found in the flow cytometry plots. To this end, conidia were incubated in liquid MM plus 1% glucose for 6 h at 37°C. Cells were harvested by shaking and centrifugation and taken up in ST. Cells were either centrifuged at 500 × *g* for 30 min through a sucrose gradient (20, 25, 27.5, 30, 35, 40, and 50% sucrose; 1 ml each) or not centrifuged. Fractions (0.5 ml) were collected and checked by microscopy. The fraction with 27.5% sucrose contained single swollen conidia. This sample was used to draw the gate (Fig. S3A and B). With this gate, we still found a few aggregates within untreated samples (Fig. S3C and D) so we index sorted single events from this gate in 96-well plates. The presence of single swollen conidia was scored per well using microscopy, and the positive events were plotted back in the flow cytometry plot. This allowed us to draw the gate for single swollen conidia with more precision (Fig. S3E and F). In total, ∼28% of the sample (6 h at 37°C) was made up of single isotropically grown conidia.

10.1128/mBio.03015-19.3 FIG S3 (A and B) Flow cytometry of enriched isotropically grown (swollen) cells in a sucrose fraction of 27.5%. Scale bar represents 10 μm (B). (C) Swollen conidia gate from panel A drawn on entire population of cells incubated for 6 h at 37°C. Note that many cells are still dormant (below the gate). Panels D and F show daughter gates of panels C and E, respectively. (D) TPW of swollen cells with the gate for single cells shown. (E) Swollen and germlings cell gate as determined by sorting. (F) TPW of swollen cells with the gate for single cells shown. Percentage of cells in each gate expressed to the total population (A, C, and E) or mother gate (D and F) are indicated. The sorting efficiency of single cells as verified by microscopy was 91%. Download FIG S3, TIF file, 2.7 MB. Copyright © 2020 Bleichrodt et al. 2020 Bleichrodt et al. This content is distributed under the terms of the Creative Commons Attribution 4.0 International license.

Conidia were incubated in liquid MM plus 1% glucose for 16 h at 30°C. To find germlings in the flow cytometry plots, we sorted events that were not single dormant or swollen conidia on microscopy slides. We found that most single germlings were present at higher TPW and low SSC (Fig. S4B). From this area, we index sorted single events in each well of 96-well plates. The presence of single germlings was scored per well, and the positive events were plotted back in the flow cytometry plot. This allowed us to draw a specific gate that predominantly contained single germlings (Fig. S4B). In total, ∼5% of the sample (16 h at 30°C) was composed of single germlings. With this method, we were able to measure cell size and fluorescence intensity of single cells of dormant and isotropically grown conidia and germlings (Fig. 1A to C, respectively). An overview of all cell types in the flow cytometry plot is presented in Fig. 2.

10.1128/mBio.03015-19.4 FIG S4 (A) The gate for swollen conidia and germlings is shown as SSC against FSC by flow cytometry. (B) The daughter gate of panel A, which includes single germlings is shown as TPW against SSC. The percentage of cells in each gate expressed to the total population (A) or mother gate (B) are indicated. The sorting efficiency of single cells as verified by microscopy was 80%. Download FIG S4, TIF file, 1.3 MB. Copyright © 2020 Bleichrodt et al. 2020 Bleichrodt et al. This content is distributed under the terms of the Creative Commons Attribution 4.0 International license.

### (ii) Flow cytometer settings.

The fluorescence levels of thousands of single dormant and isotropically grown conidia or germlings were measured by flow cytometry (see Data Set S1 for the numbers of observations). We used a BD Influx system with BD FACS Sortware 1.2.0.142 software, firmware version 7.5.1.3.16. The nozzle size was 140 μm, and the sheath pressure was 5.0 lb/in^2^. Alternatively, a Moflo Astrios EQ (Beckman Coulter) in combination with Summit V6.2 software or a BD FACSCanto II with BD FACSDiva software was used.

### Cell culturing.

Cell culturing is described in Text S1 in the supplemental material.

### Phagocytosis assay. (i) Macrophage infections.

A total of 1 × 10^6^ conidia of strain MFIGRag29, either stained with 25 nM CFW or not stained with CFW was mixed in 2 ml prewarmed (37°C) Dulbecco modified Eagle medium (DMEM) supplemented with 10% fetal bovine serum (FBS) and 1% penicillin-streptomycin (Pen-Strep) in order to obtain a multiplicity of infection (MOI) of 5. The medium of the macrophages was aspirated, and the dormant conidial suspension was added to the wells. Next, the 24-well plate (ibiTreat; tissue culture treated; Ibidi) was centrifuged at 100 × *g* for 5 min to ensure that all conidia were in close contact with the macrophages to allow phagocytosis. Coincubation was performed for 60 min at 37°C at 5% CO_2_. Thereafter the cultures were fixed with 3% formaldehyde in phosphate-buffered saline (PBS) for 15 min. Wells were washed three times with PBS and stained with CellMask Deep Red (1 μl/ml in PBS; Thermo Fisher Scientific) for 10 min at room temperature (RT). The wells were then washed again three times with PBS.

### (ii) Imaging of macrophage infections, image processing, and data analysis.

Imaging of macrophage infections, image processing, and data analysis are described in Text S1 in the supplemental material.

### Statistical methods.

For germination experiments and analysis of CFW and lectin heterogeneity labeling by flow cytometry, at least two replicates of thousands of cells were produced. For CFW-labeled conidial heterogeneity flow cytometry experiments, three replicates of generally 10,000 cells were generated. Check Data Set S1 for individual values.

### (i) Determining the level of heterogeneity through the integrated squared error between a single normal density and a mixture of normal densities and finding the “best” mixture density models for sample sets.

Briefly, the Bayesian information criterion (BIC) (37) values were calculated on log-transformed data for a mixture of up to seven normal distributions and minimized to find the best normal mixture densities for each sample. We used the d statistic (38) to describe the level of heterogeneity by the integrated squared error (ISE), which is described in detail in Text S1 in the supplemental material.

### (ii) Determining bootstrap confidence intervals for the parameters.

Determining bootstrap confidence intervals (CI) for the parameters is described in Text S1 in the supplemental material.

### (iii) Other statistical tests used.

Correlation was calculated using the Pearson correlation coefficient. For phagocytosis experiments, the Mann-Whitney U test was used to determine whether conidia had different CFW intensity levels when internalized compared to external conidia. This test was used, since data were continuous but not normally distributed even after log transformation. An independent *t* test was used to test whether CFW labeling had an effect on phagocytosis of the MFIGRag29 strain.

### Data availability.

Codes in R and ImageJ used for data and image analysis are available on Github (https://github.com/rbleichrodt/Conidial-heterogeneity.git).
