# Supplementary figures and images for: Cell Wall Composition Heterogeneity between Single Cells in Aspergillus fumigatus Leads to Heterogeneous Behavior during Antifungal Treatment and Phagocytosis
Source: mBio. 2020 May 12;11(3):e03015-19. doi: 10.1128/mBio.03015-19 (PMC7218287; doi:10.1128/mBio.03015-19)

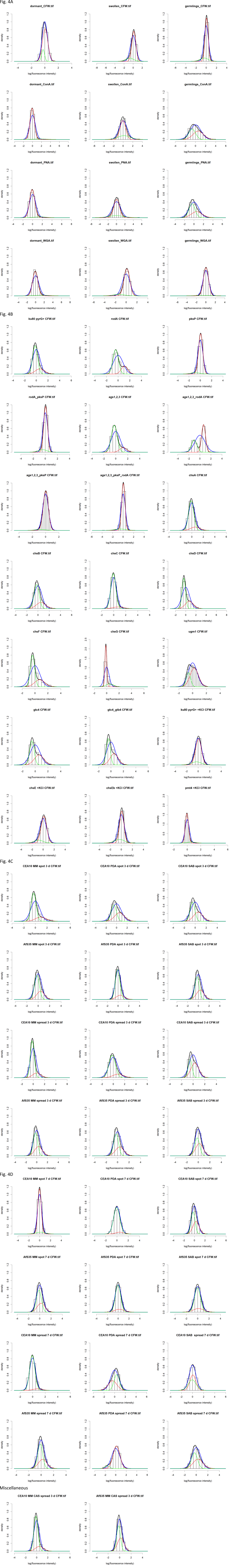

Supplement: FIG S1 [file mBio.03015-19-sf001.tif]

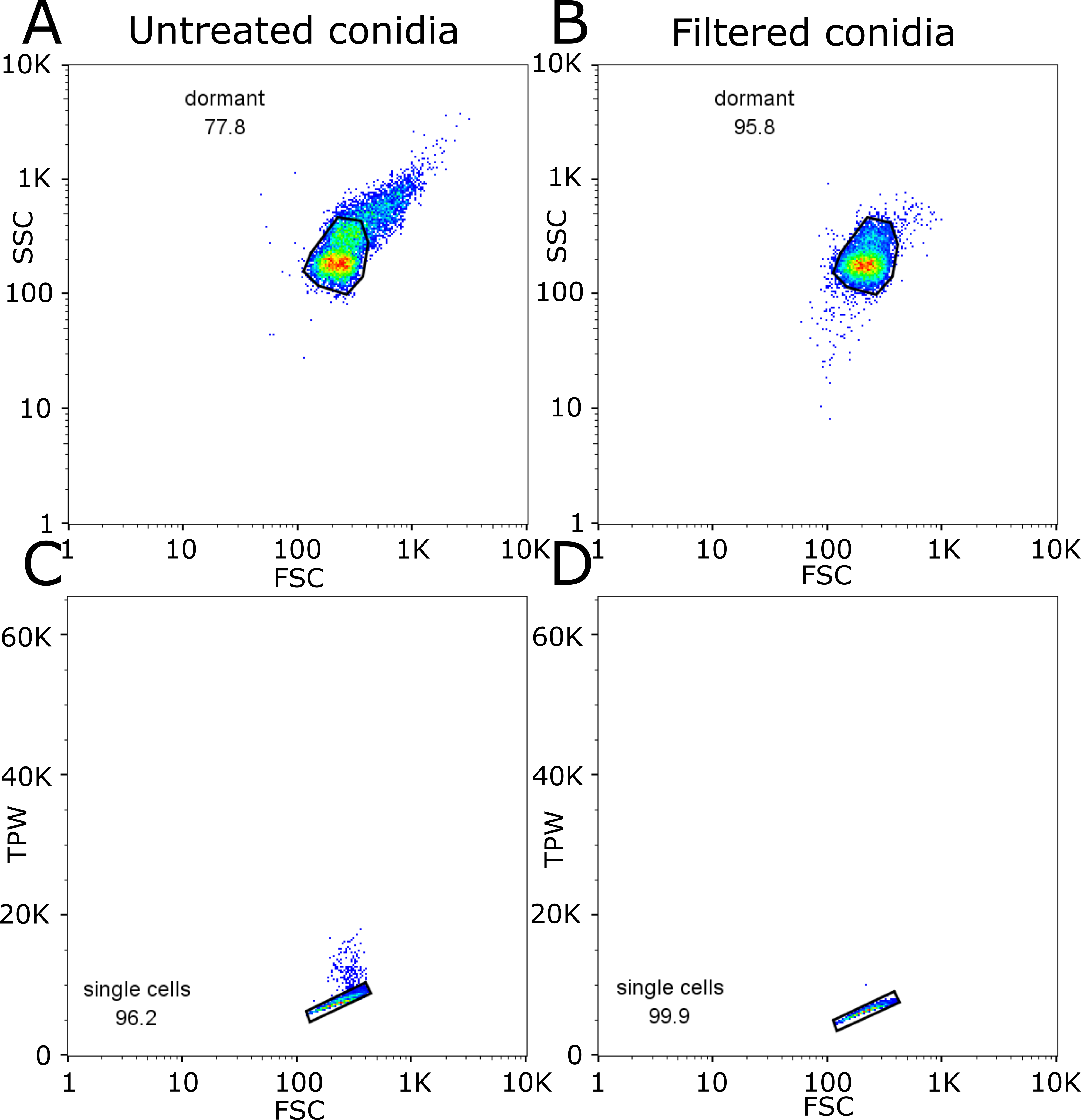

Supplement: FIG S2 [file mBio.03015-19-sf002.tif]

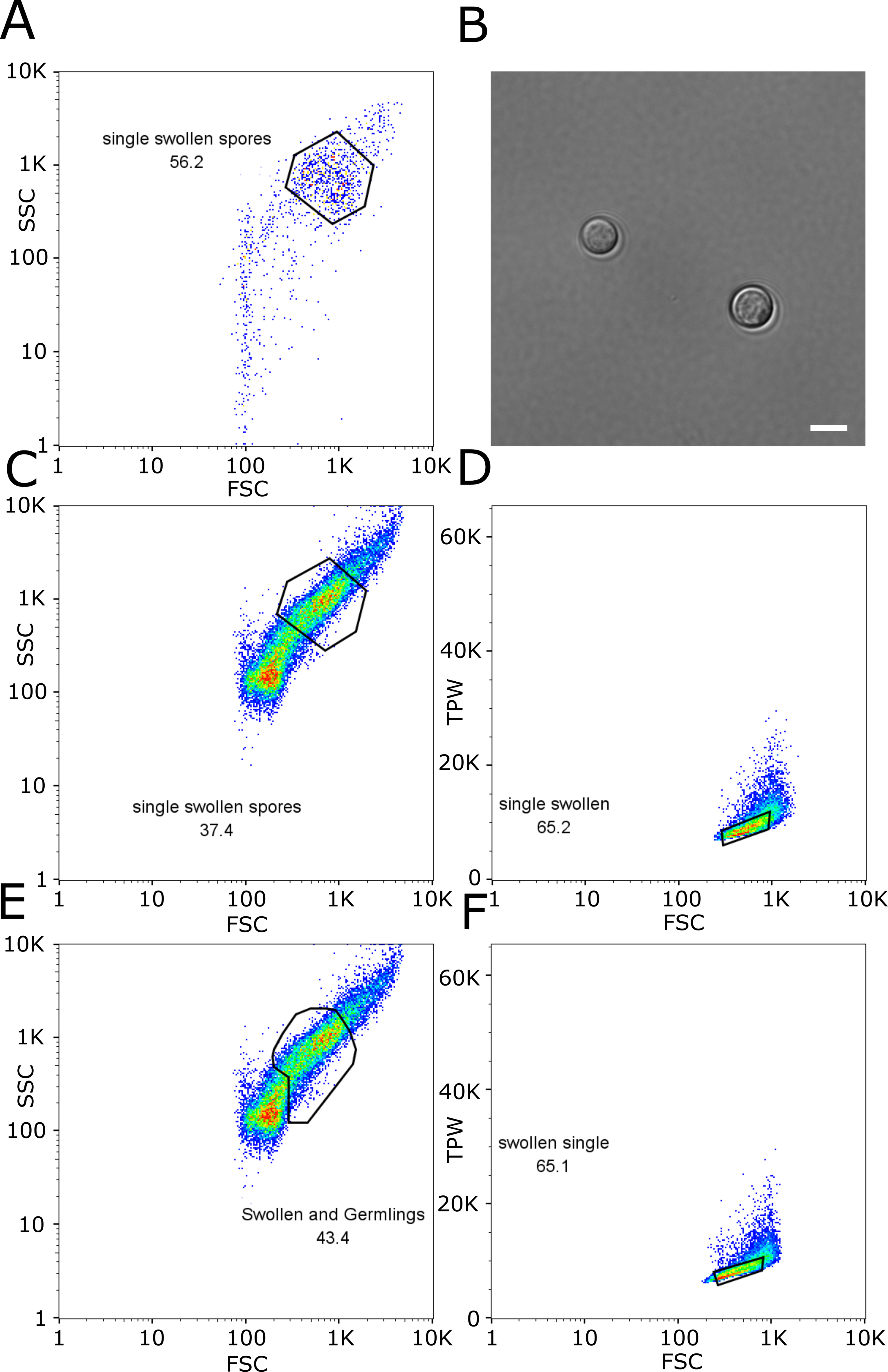

Supplement: FIG S3 [file mBio.03015-19-sf003.tif]

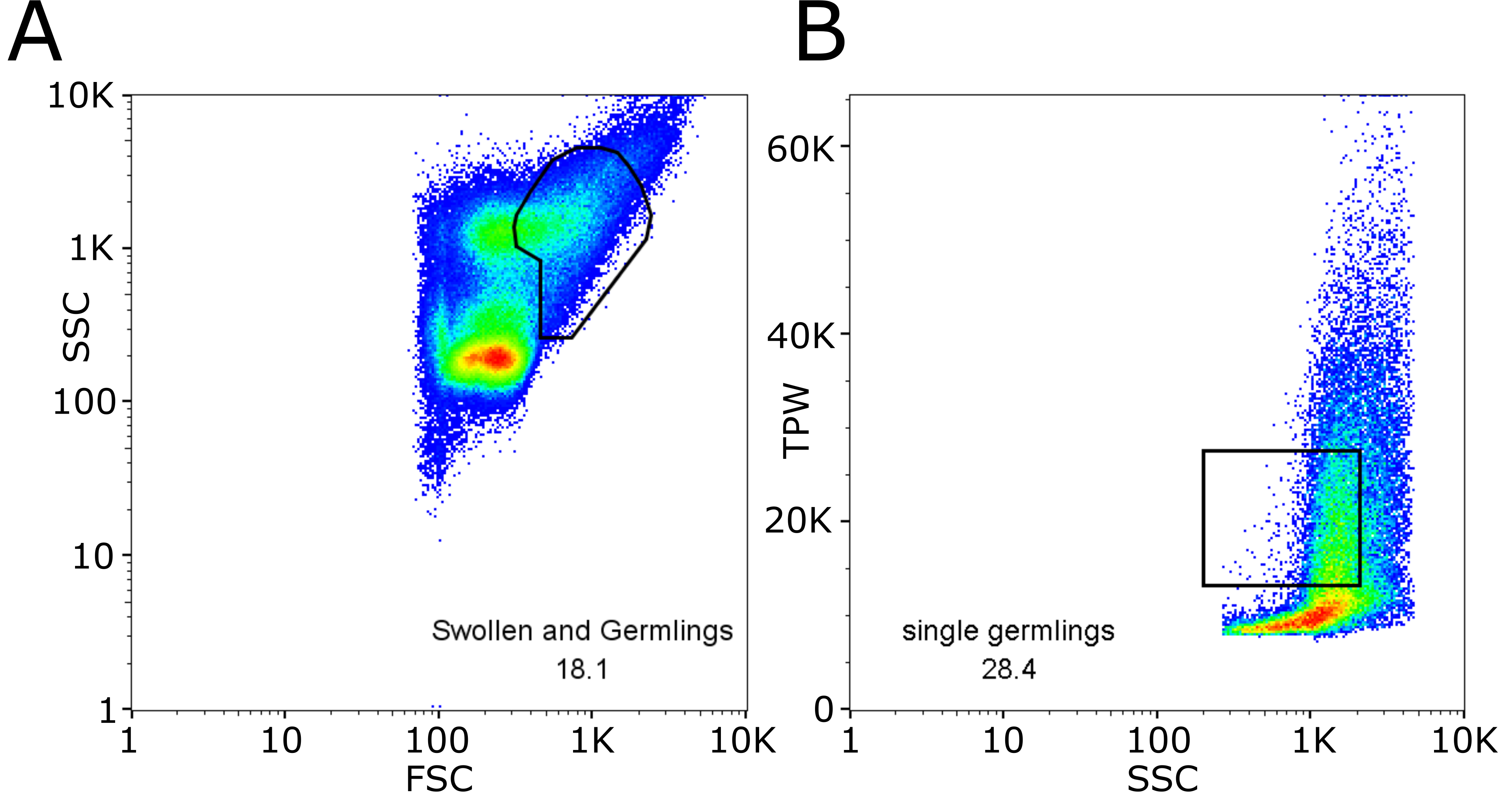

Supplement: FIG S4 [file mBio.03015-19-sf004.tif]
