# Supplementary material for: Cell Wall Composition Heterogeneity between Single Cells in Aspergillus fumigatus Leads to Heterogeneous Behavior during Antifungal Treatment and Phagocytosis
Source: mBio. 2020 May 12;11(3):e03015-19. doi: 10.1128/mBio.03015-19 (PMC7218287; doi:10.1128/mBio.03015-19)
Supplement: TEXT S1 [file mBio.03015-19-s0001.docx]

*TEXT S1: Supplemental Materials and Methods*

***Fluorescent labelling of conidia***

5*10^6^ dormant conidia ml^-1^ were suspended in ST. For swollen conidia or germlings, 1 ml samples of standing cultures were centrifuged for 10 min at 5000 rpm and taken up in ST. Cells were labelled with the lectins Wheat Germ Agglutinin(WGA)-Alexa-488 (0.5 µg/ml), Peanut Agglutinin(PNA)-Alexa-594 (1 µg/ml), or ConcanavalinA (ConA)-Alexa-488/647 (10 µg/ml) (Thermo Fisher Scientific), or with Calcofluor White (CFW; Fluorescent Brightener 28; 25 nM for microscopy and 250 nM for flow cytometry; Merck) for 10 min at RT. A stock solution of CFW (10 mg/ml) was prepared in 0.1 KOH. Cells were centrifuged for 5-10 min at 5000 rpm, either fixed with 3.7% formaldehyde in PBS, or not and taken up in ST and kept on ice in the dark. Labelling was visualised using confocal microscopy as summed intensity projections of Z-stacks (Fig. 1D-G), and analysed by flow cytometry.

***Monitoring CFW and Katushka fluorescence during caspofungin treatment***

The CFW labelling of cells of strain MFIGRag29 and cytosolic Katushka levels within cells were monitored using Confocal Laser Scanning Microscopy (CLSM) on a Leica SP8 system with a tunable white light laser (for Katushka), a 405 nm diode laser (for CFW) and a HC PL APO CS 40x/0.85 DRY objective. Katushka was excited at 555 nm (6% laser power) and fluorescence captured at 564-776 nm (gain 55.2); CFW was excited at 405 nm (0.5% laser power) and fluorescence captured at 410-547 nm (gain 159.4) at 400 Hz scan speed, with pinhole size of 99.9 µm using gating (Gate start: 0.3 ns, end: 6 ns, reference wavelength: 555 nm). Images were captured every 34 min. 26 sec at a XYZ resolution of 310 nm in an environmentally controlled microscope incubation chamber at 37 °C. To this end, multiple positions in XYZ were defined and then imaged in each sample over time. 115 conidia in the control and 136 conidia in the caspofungin (CAS) treatment were analysed, respectively. Image analysis was performed using a bespoke script in ImageJ, which produced the summed intensity of all Z-slices. The Katushka signal was used to draw a region of interest (ROI) around each cell over time and its area was measured. This was to find the time point when a cell had burst (cytosolic Katushka fluorescence is lost upon cell lysis) and analyse the growth rate. To this end, the image was blurred and subsequently thresholding was done, to produce a binary image. The particle analysis tool was then used to automatically draw ROIs over time (see Data availability for the code). This ROI set was then copied to the corresponding CFW signal to measure the average fluorescence intensity of each cell over time. Alternatively, the CFW channel was directly used to draw ROIs and measure CFW signals over time. If a cell was missing for a time point the value of the nearest time point was copied to this position to avoid gaps that would have been incompatible with data analysis. For comparison of subpopulation parameters, the value of the control condition was duplicated for every 2^nd^ time point of the caspofungin treatment (and triplicated for the last time points), since it took twice as long for the latter condition (25 time frames) to reach the germling state as the control condition (12 time frames).

***Cell culturing***

RAW264.7 murine macrophages were routinely grown on Dulbecco’s Modified Eagle Medium (DMEM) + 10% Fetal Bovine Serum (FBS) + 1% Penicillin-Streptomycin (Pen-Strep) and passed twice per week in fresh medium. Cells were used until passage 20. For phagocytosis experiments 2*10^5^ cells/well were seeded in 24-well clear bottom dishes (µ-Plate 24 Well TC treat; Ibidi). After incubation at 37 °C at 5% CO_2_ for 18 h, cultures were used for phagocytosis assays.

***Phagocytosis assay***

*Imaging of macrophage infections*

CellMask Deep Red staining of macrophages and cytosolic expression of Katushka in conidia was imaged on a Leica SP8 laser scanning confocal microscope using a HC PL APO CS 40x/0.85 DRY objective and Leica HCS A Matrix Screener software. At one position per well tiles of 900 µm x 900 µm were set. The Z-focus was determined using the autofocus function and Z-stacks were produced with an optical XYZ resolution of 310 nm. Confocal settings were: 405 nm laser power 1%, 560 nm 6% and 652 nm 0.3%, pinhole 84 µm, filters and detector gain were: 410-550 nm, gain 114; 570-631 nm, gain 770; 668-784 nm, gain 20.7. Sequential imaging of CellMask Deep Red and Katushka fluorescence was employed to limit bleed through. Typically a few hundred macrophages where imaged and analysed per treatment.

*Image processing and data analysis*

Imaris (Bitplane) was used for image analysis. First Leica (*.lif) files were batch converted to Imaris (*.ims) files. CellMask Deep Red and Katushka fluorescence was used to segment the macrophages and conidia as ‘surfaces’ and ‘spots’, respectively. To this end, the optimal image processing parameters were manually tested. These were then applied to all data sets using batch processing. This generated 3D models of macrophages represented as surfaces and fungal conidia represented as spots (Fig. 6A). Each spot was then automatically counted to determine the total number of conidia contained in the data set for each well. Each 3D model image was then finally processed using an Imaris XTension function called ‘Spots Split Into Surface Objects’ (http://open.bitplane.com/tabid/235/Default.aspx?id=19), which tested for every spot (*i.e.* conidium) whether it was inside a surface (*i.e*. macrophage) or not. This produced a subpopulation of spots that represented internalised conidia. The pool of non-internalised conidia was determined by subtracting the pool of internalised conidia from the total population of conidia, using their unique XYZ coordinates. The CFW intensity of each conidium in both the internalised and non-internalised pools was exported as an Excel file. The percentage of phagocytosis was calculated by dividing the number of internalised conidia by the total number of conidia.

***Statistical methods***

*Determining the level of heterogeneity through the integrated squared error (ISE) between a single and a mixture of Normal densities; finding "best" mixture density models for sample sets*

Data of each population was log transformed and normalised by subtracting its weighted mean (= µ_1_ * λ_1_ + µ_2_ * λ_2_) from each individual observation and biological replicates were combined for statistical analysis. First the Bayes Information Criterion (BIC; 1) values were calculated on this log transformed/normalised data for a mixture of up to seven normal distributions and minimized to find the best normal mixture densities for each sample.

$$BIC\left( m \right)= -2*\log\mathrm{likelihood}+\left( 3*m-1 \right)*\log n$$

where *m* is the number of normal densities in a mixture and *n* is the sample size. The −2∗log-likelihood part is a measure of goodness-of-fit and the additional term (3∗*m*−1)∗log *n* penalizes the fit as it is based on using 3 ∗ *m* − 1 parameters for a sample of size *n*. Minimizing over m for a single sample finds the best trade-off between the goodness-of-fit and the number of densities used in the mixture.

We used the d-statistic (2) to describe the level of heterogeneity by the integrated squared error (ISE) as follows: let $x_{1},\ldots,x_{n}$ be a random sample which is assumed to come from the Normal mixture density function given by $\sum_{j=1}^{m} p_{j}f(x|\mu_{j},\sigma_{j})$ where each $p_{j}\geq0$ and $\sum_{j=1}^{m} p_{j}=1$. Note that we can express $p_{m}=(1-\sum_{j=1}^{m-1} p_{j})$, so that there are actually only $m-1$ mixing proportions which need to be identified. Note that the Normal density $f(x|\mu_{j},\sigma_{j})=\frac{1}{\sqrt{2\pi\sigma_{j}}}\exp\left( -(x-\mu_{j})^{2}/2\sigma_{j}^{2} \right)$ for $-\infty<x<\infty$. Let $f(x|\mu_{0},\sigma_{0})$ denote the single Normal density function with mean $\mu_{0}$ and variance $\sigma_{0}^{2}$. Let $\hat{\mu}_{0}$ and $\hat{\sigma}_{0}$ denote estimates of $\mu_{0}$ and $\sigma_{0}$, respectively, calculated from the sample data (3). We typically used the sample mean and the sample standard deviation. Let $(\hat{p}_{j},\hat{\mu}_{j},\hat{\sigma}_{j}),j=1,\ldots,m$ denote the maximum likelihood estimates of the parameters in the mixture Normal model calculated using the expectation maximization (EM) algorithm. The calculations of these parameter estimates were performed using the function *normalmixEM* in the package called *mixtools* which has been developed for use with the R statistical computing software (4). The d-statistic (2) giving the integrated squared error between the two fitted (estimated) probability models for the data is: $d(m,n)=\int_{-\infty}^{\infty} \left( \sum_{j=1}^{m} p_{j}f(x|\hat{\mu}_{j},\hat{\sigma}_{j})-f(x|\hat{\mu}_{0},\hat{\sigma}_{0}) \right)^{2}dx$ $=\int_{-\infty}^{\infty} \left( \sum_{j=0}^{m} p_{j}f(x|\hat{\mu}_{j},\hat{\sigma}_{j}) \right)^{2}dx$ where $\hat{p}_{0}=-1$. When we are considering the integrated squared error, as in $d(k,n)$, it turns out that its value only depends on the parameter estimates and can be expressed exactly as:

$d(m,n)=\sum_{j=0}^{m} \sum_{k=0}^{m} \frac{\hat{p}_{j}\hat{p}_{k}}{\sqrt{2\pi(\hat{\sigma}_{j}^{2}+\hat{\sigma}_{k}^{2})}}\exp\left( -\frac{(\hat{\mu}_{j}-\hat{\mu}_{k})^{2}}{2(\hat{\sigma}_{j}^{2}+\hat{\sigma}_{k}^{2})} \right)$

### *Determining bootstrap confidence intervals (CI) for the parameters*

For a given set of sample data of size $n$, we have modelled its underlying density function by the Normal mixture model given by $\sum_{j=1}^{m} p_{j}N(\mu_{j},\sigma_{j}),$ where the parameter estimates are obtained via the EM algorithm and $m$ is chosen using a BIC (1). To gauge the uncertainty in the parameter estimates, as well as the ISE measure $d(m,n)$ defined earlier, it is useful to construct confidence intervals for their true (but unknown) values. To find such intervals we have used a parametric bootstrap approach. Efron and Tibshirani (5) claim that such methodology should give similar results to those that could be obtained if we knew the theoretical sampling distribution of the parameter estimators. However, the latter is difficult to obtain using likelihood theory in the present context. By conducting a parametric bootstrap, we are making the assumption that the fitted mixture model is the true state of nature and our bootstrap data is then a random sample obtained from this distribution. The sets of parameter estimates calculated by fitting the above model to successive bootstrap samples then enables us to build up empirically an estimate of the joint sampling distribution of the estimators for each of the parameters. This is a $(3m-1)$-dimensional distribution. To simplify matters though, rather than estimating a joint high dimensional confidence region (*e.g.* $3m-1=9$ when we include $m=3$ densities in the mixture), we have estimated each confidence interval separately using the marginal bootstrap distributions. To this end, we used $1000$ bootstrap samples (each of size $n$) and 95% confidence intervals were constructed for each true parameter value (and the ISE) using the percentile bootstrap confidence interval method. This is described in detail by Carpenter and Bithell (6) in their review of various bootstrap confidence interval methodology. The percentile method has been found to work well, provided that the sampling distribution of the estimator is not too asymmetric (5).

*Supplemental references*

1. Schwarz GE. 1978. Estimating the dimension of a model. Ann Stat 6:461-464.
2. Charnigo R, Sun J. 2004. Testing Homogeneity in a Mixture Distribution via the L2 Distance Between Competing Models. J Am Stat Assoc 99:488-498.
3. McLachlan GJ. 1988. Mixture Models: inference and applications to clustering. Statistics: Textbooks and Monographs, Dekker.
4. R Core Team. 2017. R: A language and environment for statistical computing. R Foundation for Statistical Computing, Vienna, Austria. 2017 URL http://www.R-project.org/
5. Efron B, Tibshirani R. 1993. An Introduction to the Bootstrap. Chapman & Hall, London.
6. Carpenter J, Bithell J. 2000. Bootstrap confidence intervals: when, which, what? A practical guide for medical statisticians. Stat Med 19:1141-1164.
